# Social psychiatry in Oxford and its ecological niche, 1959–1988

**DOI:** 10.1192/bjb.2024.44

**Published:** 2024-08

**Authors:** Rob Poole

**Affiliations:** Bangor University, Bangor, UK

**Keywords:** History of psychiatry, therapeutic community, social psychiatry, regulatory culture, mental health services

## Abstract

This paper reflects on a special edition of the journal *History of Psychiatry* and a related symposium held at Somerville College, Oxford, exploring the innovations in mental healthcare in Oxfordshire led by Dr Bertram Mandelbrote between 1959 and 1988. I draw on clinical culture, biography, mental health policy and my lived experience to understand Mandelbrote's life and work, and his legacy and lessons for contemporary psychiatrists. I explore the ecological niche that Mandelbrote created and conclude with the probable importance of his relationship with Professor Michael Gelder, who led the University of Oxford Department of Psychiatry at the time.

A special issue of the journal *History of Psychiatry* (HoP) was published in spring 2023. It examined the innovations that occurred in mental health services in Oxford between 1959 and 1988 under the leadership of Dr Bertram Mandelbrote.^1^ It was followed on 23 October 2023 by a symposium (Innovation in mental healthcare symposium: ‘What is to be learned from the Oxford experience?’) at Somerville College, Oxford, to mark what would have been Mandelbrote's 100th birthday.

*Mind, State and Society: Social History of Psychiatry and Mental Health in Britain 1960–2010*^2^ (available open-access online) was published by Cambridge University Press in 2021. This book, edited by Ikkos and Bouras, is the most comprehensive account of the period that is currently available. However, Mandelbrote is absent from the text, index and references, and there is no exploration of the radical innovations in social psychiatry that occurred in Oxfordshire under his leadership. Edward Shorter's older history, *A History of Psychiatry: From the Era of the Asylum to the Age of Prozac*,^3^ also omits Mandelbrote and the Oxford innovations, despite reference to several other British psychiatrists.

This absence from the standard histories of psychiatry is curious. The special issue of HoP and the Oxford symposium served to underline the extent of Mandelbrote's influence at its zenith. His impact was felt as far as Boulder, Colorado, where developments in community psychiatry led by the late Dick Warner were greatly affected by Warner's time working with Mandelbrote at the Phoenix Unit in Oxford.^4^ Warner was the author of a seminal text on social psychiatry, *Recovery From Schizophrenia*.^5^

In this article, I draw on a range of themes from the HoP special issue and the associated symposium. I touch on clinical culture, mental health policy, biography and my personal lived experience to understand Mandelbrote's legacy and lessons for modern psychiatrists.

## Bertram (‘Bertie’) Mandelbrote

Bertram Mandelbrote was born in Cape Town, South Africa, and trained in medicine at Groote Schuur Hospital. He arrived in Oxford in 1946 as a Rhodes scholar. After a period of research into Wilson's disease and initial training as a physician, he decided on a career in psychiatry. He worked at the Maudsley Hospital under Aubrey Lewis and at Warlingham Park Hospital in Surrey, where the physician superintendent, T. P. Rees, was a pioneer of open doors and humane, liberal hospital care.

The role of ‘physician superintendent’ (sometimes ‘medical superintendent’) was established during the great asylum-building era of the 19th century. It reflected an Enlightenment belief that the care of people with mental illness should be led by science. These doctors were the statutory heads of their hospitals. They held enormous power and had control over everything, both clinical and managerial. Although they had external accountability, they were to all intents and purposes autocrats, within closed communities that sometimes contained 2000 patients and staff. They were provided with large houses within the hospital grounds and domestic help from among the recovered or recovering patients. Food was supplied to them from hospital farms. They influenced all aspects of asylum life and they could make decisions that had profound ramifications for patients and staff alike. Their statutory powers were removed by the Mental Health Act 1959, and the residual role was largely abolished in the course of the 1960s and 1970s; however, this did not occur in a single step. Most large mental hospitals continued to have a dominant medic in *de facto* control of them until the general management changes that followed the Griffiths National Health Service (NHS) Inquiry of 1983.^6^ The coup de grace for medical control was the mental hospital closures of the 1990s.

Mandelbrote was appointed physician superintendent at Coney Hill and Horton Road hospitals in Gloucester at the unusually early age of 33. When he arrived, these hospitals were archaic and largely unaffected by modern thinking about mental healthcare. In characteristic style, Bertram led a series of rapid and radical changes. Doors were opened, the local community was invited in and patients who were reasonably well were discharged. Ward regimes were reformed around group activities that were both therapeutic and democratising. After 3 years of transformational change, Bertram moved to a similar post at Littlemore Hospital in 1959, taking some staff from Gloucester with him. He remained at Littlemore until his retirement in 1988. Bertram Mandelbrote died in 2010.

## The changes that Mandelbrote led in Oxfordshire

The changes that Mandelbrote initiated continued and developed throughout the rest of his career. They are described and analysed in the special issue of HoP, in papers that are valuable to anyone with an interest in the modern history of psychiatry. I was particularly interested by Armstrong and Agulnik's paper about the role of ‘happenstance’ in what took place in Oxford,^7^ with its elements of serendipity and creative opportunism. They suggest that there was an ecological niche that allowed the bottom-up developments at Littlemore (and elsewhere) to flourish; the corollary is that this niche disappeared with changes in the mental healthcare environment, particularly in the regulatory culture.

The Oxfordshire innovations fell into several broad categories:

- reform of the in-patient areas, with the introduction of a therapeutic community model into acute care (the Phoenix Unit);
- deinstitutionalisation and radical reductions in long-stay beds, facilitated by the development of a network of group homes that were managed at arm's length from the hospital;
- the establishment of social rehabilitation in the community through a social enterprise;
- the development of a distinctive model of rehabilitation for drug and alcohol addiction, focused on abstinence;
- the establishment of accessible psychotherapy services in the community.

From April 1986 to October 1987, I worked at the Phoenix Unit at Littlemore Hospital as senior registrar to Bertram. I was his last senior trainee and came to know him well. He was a rather shy man who never raised his voice. Nonetheless, his presence was pervasive throughout the hospital. He still used the title ‘physician superintendent’ in his letters, although the formal role had gone many years before. Such was his gravitas that this went unchallenged.

I think that Bertram was driven by humanitarian concerns, but he was a complex man who contained many paradoxes. One could not doubt his commitment to people who were socially marginalised, including those whose mental illness or addiction was associated with criminality. He was willing to take risky patients that other consultants declined to admit, at a time when the Oxford NHS region had no locked ward and no medium secure unit. However, Bertram did not have the common touch. He encouraged staff to abandon uniforms and all indicators of status or hierarchy, but he always wore a suit.

Most of his innovations depended on him attracting unusually able colleagues. Dr Benn Pomryn was one such. He was a consultant psychiatrist who was totally absorbed in fostering the therapeutic community regime at the Phoenix Unit. He published very little and had no interest in committees. He was one of the first to conduct psychiatric out-patient clinics in general practice, possibly following Maxwell Jones's community developments in Scotland.^8^ In the case of the Ley Community (substance misuse rehabilitation) and the Isis Centre (community psychotherapy), Mandelbrote's main partner was Dr Peter Agulnik, who worked with him from 1969.

Bertram was neither a brilliant theorist nor a charismatic clinician. He published around 25 papers and co-edited a textbook of psychiatry,^9^ a respectable but far from prolific academic output. Instead, he was a talented medical manager of great vision. He created environments where innovations could flourish. He carried enormous authority, which he deployed, for the most part, with creativity and imagination. A particular and unusual feature of his managerial approach was the regular but agenda-less meeting. He was unfailingly loyal to the colleagues and staff that he trusted.

The models of care that developed under Mandelbrote's leadership were dramatically less controlling and prescriptive than both those that went before and those now dictated by modern guidelines and regulation. Although the services were not perfect (abuses could and did occur), they aimed to help people to regain agency and autonomy in the face of mental illness or addiction.

The most enduring impact of working with Bertram on my clinical practice was his insistence that, wherever possible, I should see patients at home with one of the Phoenix Unit nurses before agreeing to admit them. The advantages in terms of quality of assessment were immediately obvious, and I believe that assessing people at home is key to good-quality community services. Bertram was not a community psychiatrist, but he played an important part in making me one.

## The downside: separateness and schism

I arrived on the Oxford Regional Higher Professional Training Scheme in psychiatry after a decade as an undergraduate and postgraduate trainee at St George's, London. I had had extensive experience of psychotherapies, which was a distinctive feature of psychiatric training at St George's at the time. Knowing nothing of the history of the Oxford mental health services, I was puzzled that Littlemore Hospital, which by 1986 was not large, was split between the consultants who ran therapeutic communities and those who seemed much more closely aligned with the Oxford Academic Department of Psychiatry. This was based in the nearby Warneford Hospital and led from 1969 onwards by Professor Michael Gelder. In fact, the division of the hospital into two parts arose 25 years earlier when Bertram's friend Dr Felix Letemendia, a biological psychiatrist, joined him at Littlemore but wanted no part in radical social psychiatry experiments. Partition was the expedient solution, but, as is so often the case, it had consequences long after the initial rationale had been forgotten.

There was a further, and more troublesome, functional schism. Bertram insisted that we ‘consume our own smoke’, meaning that his patients were never admitted anywhere other than the Phoenix, but equally, no patients ‘belonging’ to any other team were admitted to our beds. It was my job to manage the bed situation. Consequently, we could always admit our patients when we needed to. It was frequently the case that the only available beds in Oxford were at the Phoenix Unit. Bertram disapproved of what he perceived to be a lack of engagement with clinical need in other teams. Consequently, he was unbending in his refusal to help out. My opinion at the time was that he was fundamentally right, but his non-negotiable stance created major difficulties for junior doctors out of hours. Similarly, in the 1980s, Bertram resisted the development of sectorised community mental health teams, because, as he saw it, they would undermine continuity of care. Bertram's intransigence caused particular anger among some members of the academic Department of Psychiatry.

Herein lies a major problem with opportunistic development of services outside a broader policy context. During the heyday of therapeutic communities, it was well recognised that aloof separateness and schism frequently developed around them. I felt a strong commitment to the Phoenix's model of care, but it was accompanied by significant discomfort that other patients could be adversely affected by our uncompromising specialness. Maxwell Jones pursued a rather different course in developing therapeutic communities into total district mental health services.^8^ In 1962, Jones returned from a period in the USA to become physician superintendent at Dingleton Hospital in Melrose, Scotland. Many of the changes he instituted were similar to the Littlemore reforms, but the whole of Dingleton Hospital was involved, and, crucially, Jones established a system of community mental health teams which were integrated with the in-patient service. Franco Basaglia, Dick Warner, Tom Burns and many others were influenced by periods working at Dingleton. The celebrated Trieste World Health Organization Collaborating Centre was built on the Dingleton model.^10^ The Oxford changes pre-dated those in Scotland, but the lack of a total district service with a community focus probably contributes to the neglect of Mandelbrote's legacy.

## Mandelbrote and mental health policy

Enoch Powell was a Tory cabinet minister who was marginalised from mainstream politics following his aggressive promotion of racist tropes about immigrants from 1968 onwards. Prior to that, he was generally regarded as a respectable Conservative intellectual. Powell served in the Macmillan government as Minister for Health from 1960 to 1963. His performance in that role was regarded as very good. He was the first to clearly articulate a policy of mental hospital closures, announcing this in his so-called ‘water tower’ speech to the annual conference of the National Association for Mental Health (now MIND) in 1961.^11^ He said that the old mental hospitals, ‘isolated, majestic, imperious, brooded over by the gigantic water-tower and chimney combined, rising unmistakable and daunting out of the countryside’, should close and that numbers of beds would halve within 15 years. He urged treatment in the community. All of this eventually came to pass. Many of those brooding water towers and forbidding asylums were listed buildings at the time of their closure, and they are now luxury flats. Such was the fate of Littlemore Hospital, renovated and renamed the St George's Park estate. One street is called Mandelbrote Drive.

Professor Hugh Freeman interviewed Powell for this journal in 1988.^12^ Powell was asked about his advisors on mental hospital closure: ‘there were two such people particularly… One was Stanley Smith, who produced a remarkable reorientation at Lancaster Moor, and the other was Bertie Mandelbrote at Littlemore who had the marvellous axiom, “if short of staff, close beds. If still short of staff, close more beds”. It is one of those rough and ready dicta, but I liked it.’

Powell clearly remembered ‘Bertie’ with some warmth, although the accuracy of his recollection is hard to judge. They evidently got on well. They were both highly intelligent radical conservatives, who believed in freedom and self-reliance. Bertram did not share Powell's racism; on the contrary, he was bitterly opposed to the apartheid regime in his country of birth. Bertram was above all optimistic about people's ability to recover if given the right environment and the opportunity to take responsibility, rather than passively receiving care. In both Gloucester and Oxford, he drove a rapid process of moving people back into the community. He fostered an expectation among those who were admitted to mental hospital that they could and would return to a normal life. He believed that people had agency in their own recovery.

## The legacy

In Oxfordshire today, Mandelbrote's legacy is most obvious in the persistence, outside the NHS, of the rehabilitation organisations that he had a hand in establishing. Conversely, there is little trace of his legacy within NHS mental health services, either in Oxfordshire or elsewhere. Modern acute care across the UK is widely regarded as atomised, inaccessible, custodial and deteriorated in quality.^13^ The Mandelbrote-era achievements in transforming mental hospital care seem distant. Within the NHS, their ecological niche has disappeared, and they now appear to be a redundant evolutionary curiosity. A lack of salience to modern managerial concerns is probably a key factor in the neglect of Bertram's work, although his limited publication record may also be relevant. Paradoxically, his influence on the policy of mental hospital closure means that he was an unwitting architect of the current configuration of services.

A neo-liberal, commodifying ethos has transformed British mental health services over the past 25 years.^14^ NHS organisations have become corporations, and they have done what corporations do, which is to standardise, measure and avoid organisational risk. Individualised, patient-orientated forms of care are incompatible with easily measured outcomes or defining unit cost per diagnostic episode. In the face of this, there have been governmental policies aimed at making services more relevant to patient need or ‘holistic’. An example is the Mental Health (Wales) Measure 2010. This legislation is unusual in that it confers rights on mental health patients that are unrelated to detention or compulsion. Within a few years, evidence emerged that the Measure was having the unintended consequence of making access to services more difficult.^15^ Although there is some evidence that the Measure has achieved some of its aims for some patients,^16^ overall it has not protected patients in Wales from the deterioration in mental healthcare seen elsewhere in the UK. Few governmental policies succeed in achieving all of their objectives, and many meet none or have perverse consequences. In my opinion, it is inevitable that a pervasive political ethos such as neo-liberalism will thwart attempts to improve services by statute where the combination creates conflicting imperatives.

The examination of Mandelbrote's innovations in the HoP special issue and in the symposium proceedings shows that services can develop organically through patients and staff working together, within the context of clear values and a supportive management structure, without costs running out of control. The result can be care that is acceptable to and helpful for patients. There was no central policy that allowed Mandelbrote to do what he did; he was enabled by the power of his role as physician superintendent and an ability to attract like-minded colleagues to work with him. These conditions are unlikely to return within the NHS, yet there is widespread dissatisfaction with present programmatic and decontextualised service models. It seems that progressive forms of mental healthcare are more able to thrive as charities or social enterprises than in today's public sector. The third sector is where they persist in Oxfordshire, and Warner's innovations in Colorado had to move out of the public sector in order to survive and grow. To me, it seems clear that we cannot passively wait for better times. We need to make our developments within systems that grant freedom to form appropriate ecological niches.

Bertram's influence on the deinstitutionalisation agenda of the 1960s may have been overlooked because deinstitutionalisation is no longer perceived as the great, hopeful movement that it seemed at the time. Its reputation as a necessary liberation has been coloured by today's lack of beds and consequent waiting lists for admission for people with severe psychosis. Thirty years ago, Kathleen Jones was one of the first social scientists to firmly state that closing mental hospitals was a mistake.^17^ At the time, her opinions seemed reactionary, but subsequently some of her views have been vindicated.^18^

We live in an era of quality improvement in mental health services, which emphasises evidence, outcomes and elimination of unacceptable variation in care. I have to confess that I have been heavily involved in some aspects of quality improvement in British psychiatry. I also must acknowledge that, like the Welsh Mental Health Measure, quality improvement has done little to slow the deterioration of mental health services. In fact, its origins in industry may have encouraged the development of atomised production-line models of care. I do not argue for a return to unfettered freedom for mental health professionals to pursue whatever half-baked pseudoscientific project they fancy, but I do believe that better services require an environment, or ecological niche, that allows therapeutic creativity to flourish.

## The symposium

The October 2023 symposium was a rich and hopeful occasion. I was a speaker, but I can honestly state that the symposium was not tainted by self-congratulation. For me, three things were striking. The first was a renewed awareness of how many people, many of them influential in their own right, had been permanently affected by their experience of working at the Phoenix Unit and other Oxfordshire therapeutic communities. The second was a welcome sense of hope that the sorry state of our mental health services is not irreversible, and that there may be a way forward – not an effort to relive past glories but to find something better in the future, probably driven from outside of the NHS.

Both of Bertram's two sons were present at the symposium, and Dr Scott Mandelbrote offered a third revelation. When I worked in Oxford, there was a widespread belief that the tension between the Phoenix Unit and the academic unit arose from Bertram being unforgiving to Michael Gelder for being appointed to the Chair instead of him. In fact, Gelder and Mandelbrote had a warm friendship. They met regularly for dinner for many years and were supportive of each other. The schism was real enough, but it did not lie between the two of them. I now realise that a critical element in the success of the Oxford innovations was a largely unknown, supportive and, in the mythology of the time, improbable friendship between Mandelbrote and Gelder. Gelder's support was probably important in maintaining an ecological niche for social psychiatry in Oxford. Happenstance indeed.

## About the author

**Rob Poole**, MBBS, FRCPsych, is Professor of Social Psychiatry, Centre for Mental Health and Society, Bangor University, Bangor, UK

## References

[1] Hall J, Armstrong N, Agulnik P, Fees C, Kennard D, Leach J, et al. The processes and context of innovation in mental healthcare: Oxfordshire as a case study. Hist Psychiatry 2023; 34(1): 3–16. 36583592 10.1177/0957154X221140736

[2] Ikkos G, Bouras N, eds. Mind, State and Society: Social History of Psychiatry and Mental Health in Britain 1960–2010. Cambridge University Press, 2021.

[3] Shorter E. A History of Psychiatry: From the Era of the Asylum to the Age of Prozac. Wiley, 1998.

[4] Fannon D. Richard Warner. Psychiatr Bull 2009; 33(6): 240.

[5] Warner R. Recovery from Schizophrenia: Psychiatry and Political Economy (3rd edn). Routledge, 2003.

[6] Gorsky M. ‘Searching for the people in charge’: appraising the 1983 Griffiths NHS management inquiry. Med Hist 2013; 57(1): 87–107. 23393404 10.1017/mdh.2012.82 PMC3566753

[7] Armstrong N, Agulnik P. Happenstance and regulatory culture: the evolution of innovative community mental health services in Oxfordshire in the late twentieth century. Hist Psychiatry 2023; 34(1): 64–77. 36533517 10.1177/0957154X221136702 PMC9902968

[8] Jones M. From hospital to community psychiatry. Commun Ment Health J 1970; 6: 187–95. 10.1007/BF01435915 5520334

[9] Mandelbrote BM, Gelder MG. Psychiatric Aspects of Medical Practice. Staples Press, 1972.

[10] Mezzina R. Community mental health care in Trieste and beyond: an ‘open door–no restraint’ system of care for recovery and citizenship. J Nerv Ment Dis 2014; 202(6): 440–5. 24840089 10.1097/NMD.0000000000000142

[11] Powell EJ. *Enoch Powell's Water Tower Speech 1961* (http://studymore.org.uk/xpowell.htm).

[12] Freeman H. In conversation with Enoch Powell (Minister of Health, July 1960–October 1963). Bull R Coll Psychiatrists 1988; 12(10): 402–6.

[13] Moore A. The forgotten foundations: in core mental health services, no one can hear you scream. BJPsych Bull 2018; 42(6): 225–8. 30295226 10.1192/bjb.2018.79 PMC6465218

[14] Poole R, Robinson CA. Breaking out of the citadel: social theory and psychiatry. BJPsych Bull 2023; 47(3): 146–9. 35289262 10.1192/bjb.2022.17 PMC10214419

[15] Poole R, Tippett M. Mental health services in Wales: policy, legislation and governance. InManagement for Psychiatrists (4th edn) (eds D Bhugra, S Bell, A Burns): 118–130. RCPsych Publications, 2016.

[16] Smothers M, Hill C, Lawrence D, Bagshaw R, Watt A. Predictors of recovery in a medium secure service: influence of the Welsh Government's Mental Health (2010) Measure. Int J Law Psychiatry 2023; 91: 101935. 37717488 10.1016/j.ijlp.2023.101935

[17] Jones K. Asylums and After. A Revised History of the Mental Health Services: From the Early 18th Century to the 1990s. Continuum International Publishing Group, 2000.

[18] Ikkos G. Kathleen Jones’ *Asylums and After. A Revised History of the Mental Health Services: From the Early 18th Century to the 1990s* (1993). Br J Psychiatry 2017; 211(6): 380. 29196393 10.1192/bjp.bp.117.201293

